# Factors in hybridization of local medical systems: Simultaneous use of medicinal plants and modern medicine in Northeast Brazil

**DOI:** 10.1371/journal.pone.0206190

**Published:** 2018-11-14

**Authors:** André Luiz Borba Nascimento, Patrícia Muniz Medeiros, Ulysses Paulino Albuquerque

**Affiliations:** 1 Laboratório de Ecologia e Evolução de Sistemas Socioecológicos (LEA), Centro de Biociências, Universidade Federal de Pernambuco, Recife, Pernambuco, Brazil; 2 Programa de pós-graduação em Botânica, Universidade Federal Rural de Pernambuco, Recife, Pernambuco, Brazil; 3 Grupo de Etnobiologia e Ecologia Humana, Centro de Ciências Agrárias, Universidade Federal de Alagoas, Rio Largo, Alagoas, Brazil; Missouri Botanical Garden, UNITED STATES

## Abstract

The presence of mainstream medicine in local medical systems inserts a set of external treatments and concepts that generate adjustments in the local conceptions of health and disease. What points in the system are most receptive to change? Who are the residents most likely to adopt these external treatments to deal with diseases? To answer these questions, this work used a study model consisting of the simultaneous use of medicinal plants and modern medicine, testing whether diseases that require greater treatment efforts are the main targets of adherence to modern medicine and if socioeconomic characteristics of residents can cause intracultural variation in relation to simultaneous use. To obtain socioeconomic data on the knowledge of medicinal plants and simultaneous use of these resources with modern medicine, semistructured interviews were conducted in a rural community that has easy access to modern medicine. Participatory workshops were held to access the local perceptions about the frequency of occurrence and severity of illnesses. A multilevel logistic regression model was applied for data analysis. We found that chronic, severe and frequently occurring diseases in the community tended to show greater simultaneous use locally. Among the socioeconomic factors, we determined that high educational levels positively influenced the combined use of plants and modern medicine. The need to ensure the cure of frequent, severe and chronic diseases is a factor that leads residents to seek a greater number of possible treatments, stimulating the combined use of plants and modern medicine. Residents with higher educational levels were more likely to use a combination of treatments than those with lower educational levels, demonstrating that more participation in formal education may facilitate the combined use of medicinal plants and modern medicine.

## Introduction

Local medical systems (LMS) are the social institutions and traditions that have been generated by the evolution of strategies linked to health promotion in small human groups [1, 2]. In a wider sense, a cosmopolitan medical system (CMS) is related to the global way of dealing with health problems [2], based on scientifically validated methods. Often, these two medical systems are not isolated from each other, which yields a high variety of treatments and concepts about health and disease in the same human population [3].

The access to CMS in small-scale societies generates changes in the ways in which residents deal with disease. Varying adjustments of local health practices to the existence of external forms of healing have characterized the medical system hybridization process [4]. This dynamic has been a source of ethnobotanical studies, in which researchers commonly evaluate whether or not the presence of modern medicine coexists with the use of medicinal plants [5–9]. However, little is known about which factors influence residents to adopt a new treatment (modern medicine) for a disease that they already know how to treat (medicinal plant). To better understand these factors, we are using a model that combines the use of modern medicine with medicinal plants in a rural community in Northeast Brazil.

In Brazil, especially in the northeast region, access to CMS has been encouraged by social programs funded by the Brazilian Health Ministry, such as the Family Health Program [10, 11]. This program has been implemented health through clinics and health agents in rural communities and distributes free modern medicines to residents who do not have the financial resources to purchase these medicines. Thus, this region is an adequate environment for understanding the hybridization process, since both resources are available.

Taking in account that dealing with disease events is a very important factor for survival [12], illnesses that require greater healing efforts may be more susceptible to the adoption of new medicinal treatments. It is known that residents who are subjected to chronic diseases tend to seek alternative therapies for their healthcare [13]. Therefore, severe illnesses (due to lethality or prolonged time of manifestation) can favor the inclusion of modern medicine into local medical systems. Moreover, diseases that occur very frequently used to have a greater number of different local treatments [14, 15]. Possibly, the constant need to deal with frequent health problems causes residents to seek new treatment methods. Thus, modern medicines capable of dealing with common diseases can easily be adopted in local medical systems because of the greater need for cures.

Another factor that may facilitate the adoption of new treatments in local medical systems is the low number of medicinal plants known to treat a particular disease (see [16]). Several studies report a major difference in relation to the number of plants known to treat different health problems in human populations [14, 15, 17, 18]. Thus, residents could adopt modern medicine for illnesses with few medicinal plant options, which would increase the diversity of treatments and would guarantee greater local therapeutic flexibility.

Another important question to address is: who are the residents more likely to use modern medicine? Some CMS concepts are not well assimilated by some residents in traditional communities because these ideas contradict local perceptions about health and disease [19]. For instance, a study by Waldstein et al. [20] showed that Mexican migrant women in the United States know both medicinal plants and modern medicine. However, they prefer to use traditional medicine because they believe modern medicine is dangerous, while the use of plants is not considered to have any side effects. Women are described in many ethnobotanical studies as responsible for family health care and thus know more about medicinal plants than men [21–23]. This close relationship between women and medicinal plants may cause them to resist adopting modern medicine.

Age is also an important factor. Several studies have reported that older residents are more knowledgeable about medicinal plants than younger generations [24, 25]. Some of these works attribute this difference to the limited interest of younger residents in the practice of traditional medicine [24]. Therefore, this lack of interest in medicinal plants by younger residents may facilitate the use of modern medicine. Education has also been related to the abandonment of local knowledge [26]. Time and resources spent by residents to learn academic knowledge instead of traditional knowledge have been indicated as the main cause of this shift [26]. In this sense, residents with higher educational levels could be more predisposed to adopting practices related to academic knowledge, such as the use of modern medicine.

Finally, another important factor to consider are the residents who are locally recognized as having considerable local knowledge about medicinal plants and as the experts [27]. Local experts tend to exhibit conservative behavior in local medical practices, using fewer CMS approaches [28]. This factor may reflect a greater resistance of this group to the use of modern medicine.

In this scenario, the present study tests the following hypotheses: H1—The simultaneous use of modern medicine and medicinal plants is driven by the need of the local medical system to deal with chronic, severe and frequent diseases. H2 –Diseases with few known local treatments are more susceptible to the combined use of medicinal plants and modern medicine; H3—The simultaneous use of modern medicine and medicinal plants is less widespread among older residents, those with lower levels of formal education, women, and local experts.

## Materials and methods

### Study site and survey participants

The community of Carão is located in the municipality of Altinho, in the state of Pernambuco, in northeastern Brazil (08°35’13.5”S and 36°05’34.6”W). This community represents a small rural population in the semiarid region of the state and is located 16 km from the urban center of the municipality. The vegetation type of the area is hypoxerophilic caatinga, characterized mainly by deciduous or semideciduous arboreal species and a high frequency of the botanical families Fabaceae, Euphorbiaceae, Cactaceae and Bromeliaceae [29].

In this locality, there is a healthcare unit where two health agents work. They are responsible for making medical appointments and distributing modern medicines to the residents. However, some medicines take too long to be made available to residents, and medical appointments do not occur very often. Thus, the main way that residents deal with health problems in this community remains the use of medicinal plants.

In the community, there is also a school that provides education though elementary school I. However, it is necessary to commute to the urban center for the continuation of studies. Data provided by the healthcare unit report the presence of 155 inhabitants in the community of Carão, which are distributed in 55 residences, and of which 101 are over 18 years old. The main activity conducted locally is subsistence agriculture, whose most common crops are corn, beans and cassava.

Interviews were conducted with all residents over 18 years old who consented to participate in the study, totaling 99 residents (age range: 19 to 88 years). Of these, 51 were female and 48 male. All participants who agreed to be part of this research were asked to sign a statement of informed consent allowing the collection, use and publication of data, as required by Brazilian legislation (Resolution No. 466, dated 12/12/2012, of the National Health Council of Brazil). The study was approved by the Research Ethics Committee Involving Human Beings at the University of Pernambuco (UPE), under the number CAAE: 64811715.3.0000.5207.

### Data collection

Data on the simultaneous use of modern medicine and medicinal plants were obtained through semistructured interviews [30]. Initially, each participant was invited to list the known medicinal plants and the health problems treated with them. We also asked about the signs or symptoms used by the informants to identify each cited illness. Then, for each association of a plant with a health problem, the informants were asked if they knew of modern medicines that cure the disease. When the answer was positive, we asked if they used the modern medicine in conjunction with the medicinal plant and the reason that led them to this simultaneous use. None of the participants cited diseases with only modern medicines known as treatment.

The socioeconomic data (sex, age and education) of each participant were also recorded. To recognize the local experts on medicinal plants, each participant was asked which member of the community he or she would consult if he or she did not know how to treat a disease. We applied the concept of attributed prestige by Henrich and Gil-White [31], which refers to the social status attributed to an individual who is recognized and respected for his or her expertise, success, wisdom or knowledge in a given area.

Upon completion of all interviews, the health problems were standardized according to the locally referenced symptoms. The names generated after the standardization were herein treated as therapeutic targets [15] and represented by symptomatic manifestations (such as pain) of complex health problems (such as pneumonia). These therapeutic targets were used for the second phase of the data collection, which consisted of obtaining the local perceptions of the community about their frequency of occurrence and severity.

Thus, all members of the community were invited to two workshops; in the first, they collectively discussed the disease’s frequency of occurrence and in the second workshop, the disease’s severity. The participatory workshop [30] consisted of the elaboration of cards with the names of the therapeutic targets (a total of 77) that were distributed to the participants who together assigned scores varying from 0 to 10 as to the frequency of occurrence and severity. To enable the participation of illiterate residents, the cards were read individually. After reading each card, there was a group discussion on which score should be assigned to each target. During the workshop, the participants were allowed to modify the score of the target, since a demonstration with a new card occasionally modified the group position on a previously made decision. To facilitate the visualization of all the notes and ensure the possibility of changes, after each decision the cards were arranged on the floor in association with the assigned scores.

These two workshops were conducted at different times. One for classification of severity and the other one for frequency. The separation of activities was intended to prevent the decisions of the first workshop from interfering with the answers of the second. Thus, 35 residents participated in the frequency workshop, while 24 residents attended the severity workshop. In both cases, the groups were mixed, formed by younger men and women (over 18 years) and older residents. The classification of the therapeutic targets as chronic or acute was based on the data available on the World Health Organization website [32].

### Plants collection and identification

Plant collection was performed with the assistance of local residents recognized as having considerable knowledge about local vegetation, according to the protocol of the guided tour technique [30]. No specific permissions were required for these activities, and the field studies did not involve endangered or protected species. The collection and processing of botanical material were implemented using the standard methodology in taxonomic studies [33]. The identification of the material collected was performed by specialists and by consulting herbaria. The material was then deposited in the collection of the Professor Vasconcelos Sobrinho Herbarium at the Federal Rural University of Pernambuco (PEURF).

### Data analysis

To investigate which factors better explain the simultaneous use of medicinal plants and modern medicine, a multilevel logistic regression was applied. For this purpose, a spreadsheet was created, in which the citations of medicinal plants were registered for each therapeutic target per participant in the interviews. Subsequently, a presence and absence column of citations was generated, where "0" symbolized the absence of simultaneous use with modern medicine, and "1" indicated the presence of simultaneous use. This dichotomous data was used as a response variable for the analysis.

We calculated the total number of plants locally cited for the treatment of each therapeutic target. This value was used as a predictive variable to test if residents were more likely to adopt modern medicine to deal with diseases with few medicinal plant options. To understand if disease characteristics could influence the adoption of modern medicine as part of the treatment, we used the following predictive variables: the local perception of the disease frequency and severity; and the manifested form of the disease (chronic or acute).

Finally, to understand who are the residents more likely to use medicinal plants and modern medicine combined, the predictive variables were: age (years), sex (male and female), education (from illiterate to university level) (Table 1) and level of prestige in the community (measured by the number of residents who indicated the participant as a reference on knowledge of medicinal plants).

**Table 1:** Schooling categories cited in Carão Community, northeast Brazil, and the respective continuous numeric values used in data analysis.

Value	Educational categories	Value	Educational categories	Value	Educational categories
0	Illiterate	3	Completed elementary school	6	Incomplete high school
1	Literate	4	Incomplete middle school	7	Completed high school
2	Incomplete elementary school	5	Completed middle school	8	College/University

To test the validity of the described model, it was compared with a null model (which considered only the effect of grouping by participants) using the X^2^ test for adjustments of the models through the ANOVA function, using the maximum likelihood estimation [34]. To determine which of the explanatory variables generated the best fit model, new models were generated, successively removing the explanatory variables included in the complete model and comparing their adjustment values using the AIC (Akaike Information Criteria) criteria.

All graphs and analyses were performed using the software R version 3.4.1 [35] with the support of the packages lme4 [36], lmerTest [37], ggplot2 [38], and sjPlot [39].

## Results

### Factors that influence the combined use of medicinal plants and modern medicine

Among the 99 participants in the study, 53 mentioned simultaneous use of medicinal plants with modern medicine, showing that the practice is well spread in the community studied. The influence of the disease characteristics (frequency, severity, form of manifestation and number of known local treatments) on this combined use was modeled (Table 2). The models generated show that the frequency, severity and form of manifestation (chronic) of the therapeutic target are the factors that explain the simultaneous use of modern medicine and medicinal plants (Frequency—AIC = 1175.4, χ2 = 11.23, p<0.001; Severity—AIC = 1178.5, χ2 = 8.13, p = 0.004; Disease manifestation- AIC = 1179.3, χ2 = 7.39, p = 0.006). However, the quantity of known medicinal plants for disease treatment does not influence the combined use (AIC = 1186.6, χ2 = 0.05, p>0.05).

**Table 2:** Multilevel logistic regression models generated for understanding the overlapping use of modern medicine with medicinal plants.

Fixed effect	Null model	Model 1	Model 2	Model 3	Model 4	Model 5
	Coefficient (standard error)	Coefficient (standard error)	Coefficient (standard error)	Coefficient (standard error)	Coefficient (standard error)	Coefficient (standard error)
Intercept	-2.51 (0.37)*	-2.56 (0.37)*	-2.53 (0.37)*	-2.65 (0.38)*	-2.51 (0.37)*	-4.91 (0.57)*
Frequency	-	0.29 (0.09)*	-	-	-	0.25 (0.05)*
Severity	-	-	0.24 (0.08)*	-	-	0.13 (0.03)*
Disease (chronic)	-	-	-	0.54 (0.20)*	-	0.64 (0.25)*
MPK	-	-	-	-	0.41 (0.21)	-0.01 (0.01)
**Random effect**	Variance (standard deviation)	Variance (standard deviation)	Variance (standard deviation)	Variance (standard deviation)	Variance (standard deviation)	Variance (standard deviation)
*Level 2*						
Participants	7.44 (2.73)	7.39 (2.72)	7.64 (2.77)	7.71 (2.78)	7.42 (2.72)	8.05 (2.84)
**Fit**						
AIC	1184.7	1175.4	1178.5	1179.3	1186.6	1146

Model 1 –evaluates the effect of disease frequency; Model 2 –evaluates the effect of disease severity; Model 3 –evaluates the effect of the form of disease manifestation (chronic or acute); Model 4 –evaluates the effect of the number of medicinal plants known (MPK) to deal with the disease; Model 5 –evaluates the combined effect of the previous factors.

*p < 0.05

**Fig 1:**
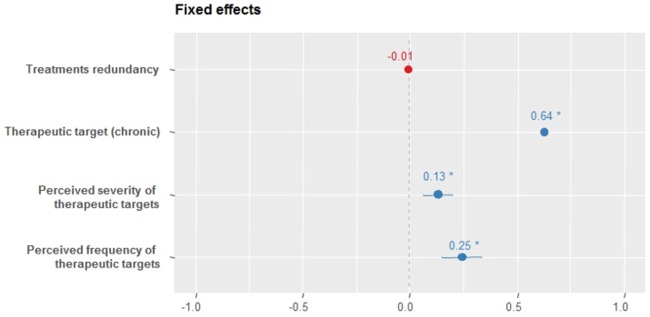
Fixed effect coefficients graph for Model 5. The Model 5 represents the insertion of disease characteristics that could explain the overlapping use of modern medicine and medicinal plants in the Carão community, northeastern Brazil. * p <0.05.

Regarding the direction of the relationship, the combination of treatments occurs for more frequent, more severe and chronic diseases (Fig 1). These three factors together better explain the combination of treatments than each one separately (AIC = 1146, χ2 = 46.69, p<0.0001). The association of treatments, therefore, is linked to health problems that require greater local treatment efforts.

The reasons expressed by the participants for the simultaneous use of plants and modern medicines are presented in Table 3. Ensuring a cure was the most cited reason (54%). The second most cited reason was the synergistic effect, that is, the addition of treatments generating an acceleration in the health improvement process (13%). If we associate these data with the results of previous models, the decision to combine modern medicine with medicinal plants is deliberate, guided by the need to guarantee the cure of some diseases.

**Table 3:** Citation percentage of the criteria used by the Carão community, northeastern Brazil, that justify the overlapping use of modern medicine and medicinal plants in the same treatment.

Criteria	Criteria meaning based on the information provided by the participants in the research	Number of citations	%
Ensure healing	The combination ensures the expected effect, because if one of the treatments fails, the other can work.	164	54
Synergistic effect	The addition of treatments accelerates healing.	38	12
Availability	Any of the treatments can be used, either together or separately, depending only on the availability of the plant and/or the modern medicines during the disease event.	25	8
Local indication	The use of the medicinal plant is advised by someone in the community, but the participant continues to use modern medicine because he or she considers it more effective.	25	8
Use of modern medicines at critical times	The plant is the most used treatment for the disease; however, in the worst cases, modern medicine is used in combination.	16	5
Experimentation	The combination of treatments is considered an efficiency test of the treatments to choose the best one to use in a future disease event.	14	5
Plant as a prophylactic	The plant is used to prevent disease. When a participant is affected by the disease, he or she begins to use modern medicine but continues to use the plant.	12	4
Medical indication	The use of modern medicine follows a doctor appointment, but the participant continues to use the plant because the medicinal plant is considered more effective.	9	3
Use of medicinal plants at critical times	Modern medicine is the most used treatment for the disease; however, in the worst cases, a medicinal plant is used in combination.	2	1
**Total**		305	100

Criteria: were generated by the researcher interpretations of the information provided by the participants during the interviews.

### Intracultural variation on the combined use of medicinal plants and modern medicine

To determine if the socioeconomic characteristics of the participants influenced the simultaneous use of modern medicine with medicinal plants, we produced some models (Table 4). The generated models show that only education explains the simultaneous use of modern medicine and medicinal plants (Education—AIC = 1172.3, χ2 = 16.24, p = 0.001). The models with age, sex and prestige did not influence the data (Age—AIC = 1181, χ2 = 2.62, p>0.05; Sex—AIC = 1184.7, χ2 = 1.99, p>0.05; Prestige—AIC = 1186.6, χ2 = 0.05, p>0.05).

**Table 4:** Multilevel logistic regression models with socioeconomical variables for understanding of the overlapping use of modern medicine with medicinal plants.

Fixed effect	Model 6	Model 7	Model 8	Model 9	Model 10	Model 11
	Coefficient (standard error)	Coefficient (standard error)	Coefficient (standard error)	Coefficient (standard error)	Coefficient (standard error)	Coefficient (standard error)
Intercept	-4.64 (2.03)*	-3.89 (0.56)*	-0.29 (1.95)	-2.10 (0.44)*	-2.54 (0.39)*	-6.39 (0.72)*
Education	0.55 (0.20)*	0.47 (0.13)*	-	-	-	0.49 (0.13)*
Age	0.01 (0.03)	-	-0.04 (0.02)*	-	-	-
Sex (male)	-0.67 (0.61)	-	-	0.65 (0.15)	-	-
Prestige	0.05 (0.09)	-	-	-	0.02 (0.10)	-
Frequency	-	-	-	-	-	0.22 (0.04)*
Severity	-	-	-	-	-	0.14 (0.03)*
Disease (chronic)	-	-	-	-	-	0.72 (0.23)*
**Random effect**	Variance (standard deviation)	Variance (standard deviation)	Variance (standard deviation)	Variance (standard deviation)	Variance (standard deviation)	Variance (standard deviation)
*Level 2*						
Participants	6.07 (2.46)	6.16 (2.48)	6.76 (2.60)	7.18 (2.68)	7.48 (2.73)	6.70 (2.59)
**Fit**						
AIC	1176.1	1172.3	1181	1184.7	1186.6	1132.6

Model 6 –evaluates the effect of all socioeconomic variables; Model 7 –evaluates the effect of education; Model 8 –evaluates the effect of age; Model 9 –evaluates the effect of sex; Model 10 –evaluates the effect of local prestige as an expert on medicinal plants; Model 11 –evaluates the combined effect of education and disease characteristics.

*p < 0.05

**Fig 2:**
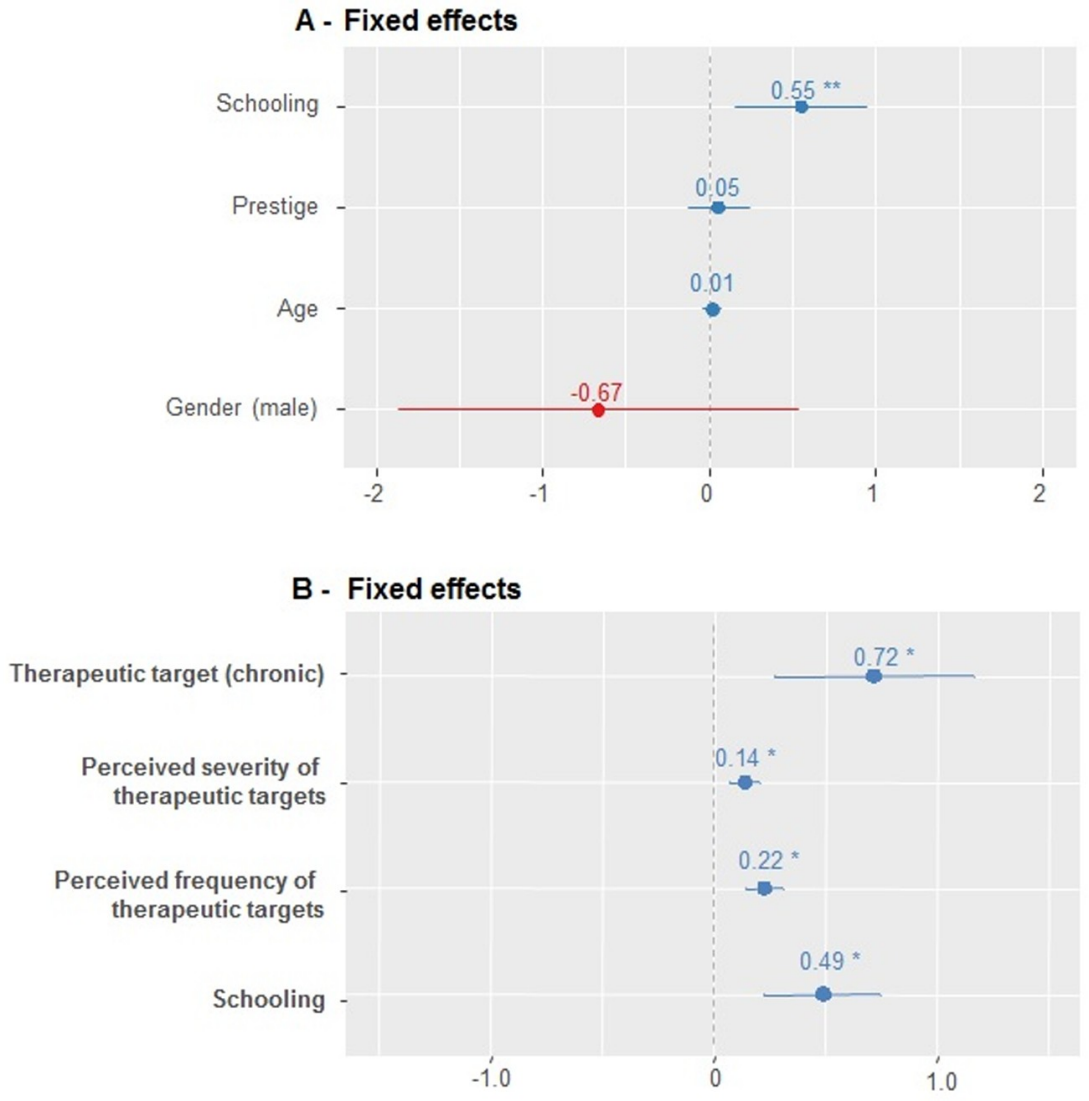
Fixed effect coefficients graph of the models 6 and 11. A- Model 6, which represents the insertion of socioeconomic variables to explain the overlapping use of modern medicine and medicinal plants; B- Model 11, which represents the insertion of socioeconomic and functional variables with a better fit to explain the overlapping use of treatments in the Carão community, Northeast Brazil. * p <0.05.

Participants with higher educational levels tend to use more modern medicines in combination with medicinal plants (Fig 2A). This finding shows that participation in formal education influences the local treatment choices. The explanatory potential of the model increases significantly (AIC = 1132.6, χ2 = 14.13, p<0.001) when the socioeconomic variable education was included in association with the variables selected in the previous model (frequency, severity and manifestation of therapeutic targets). Thus, it is possible to conclude that the association of socioeconomic and functional factors is modeling resident decisions about the combined use of modern medicine and medicinal plants (Fig 2B).

## Discussion

The findings of the present study show that some diseases are more likely to be treated with the combination of modern medicine and medicinal plants than others. One of the factors that drives residents to use both treatments together is a disease’s frequency of occurrence as perceived by the residents in the community. This evidence suggests that residents tend to seek as many treatments as possible to deal with the most frequent local health problems.

This result shows that the decision to combine treatments follows adaptive reasoning. Disease events are disorders that affect the body’s natural state of balance [40]. Therefore, residents who are capable of dealing with these events are more likely to survive. Frequently occurring health problems are a constant threat, which may compel residents to search for more efficient and rapid cures. This circumstance is especially important in rural areas where the avoidable death rates are high, and even common illnesses, such as work injuries, are more serious and severe than in urban areas [41].

Mathez-Stiefel et al. [9] conducted an interesting study about the use of traditional medicine and biomedical healthcare in Andean communities. In this research, the first therapeutic strategy was the use of medicinal plants. However, some of the interviewees said they adopted going to medical posts as a secondary health option, mainly in cases of “known” diseases (e.g., common cold, fever, pain, headache, stomach ache, and tooth ache) [9]. Although the authors did not explain what “known” disease means, according to the examples cited, this term may be associated with frequently occurring diseases. This result corroborates with our findings that the frequency of the disease influences the use of modern medicine.

In addition to frequency, severe and chronic diseases showed an important relationship with the combination with modern medicine. Again, this trend may be explained by the necessity to improve the local remedies to treat disease, since the intensity of the disorder is also a factor that compromises human survival. The participant responses about the reasons that lead them to use both resources in the treatment of the same disease support this idea, since the main reason cited is the need to ensure the disease cure, regardless of the effect originating from a plant or modern medicine.

This tendency to search for different treatments to ensure the cure of chronic diseases is also seen in patients attending hospitals and clinics in large urban centers, who seek complementary alternative therapies for their healthcare in these situations [13]. In a study by Mathez-Stiefel et al. [9], some of the interviewees mentioned seeking new forms of healing (with indigenous specialists or at medical posts) when self-treatment is not sufficient to deal with serious diseases. This result is related to sequential, not simultaneous, use but contributes to our line of discussion in that the need to cure severe diseases facilitates the adoption of a new treatment. This behavior may reflect the fact that information whose content is relevant to human survival tends to be adopted by human populations because of its adaptive potential [42, 43].

However, some studies have shown that in other rural communities in Brazil, residents used to know of few medicinal plants to deal with severe diseases [14, 15]. These studies convey the idea that residents would avoid trying new treatments for severe diseases due the high risk of death associated with failure during the experimentation process. Thus, why do residents behave differently in the use of modern medicine? This difference probably occurs because the use of modern medicine is associated, typically, with doctor recommendation. Residents tend to more easily adopt a behavior or attitude displayed by individuals they recognize as prestigious [31]. In this context, the risk associated with treating a severe disease with a new treatment is underwhelmed by the trust in physician knowledge. Therefore, the increasing doctor presence due to government programs in Brazil, such as the previously cited Family Health Program [11], may influence the dissemination of modern medicine use.

A similar program (although with emphasis on respiratory disease) developed in rural areas in Australia showed high engagement levels by the indigenous residents due the presence of doctors in their community [44]. This kind of engagement may also happen in the community studied, which contributes to explaining the combined use of medicinal plants and modern medicine. However, we have not directly investigated how these policies affect the therapeutic strategies in the community, which is an interesting issue for future studies.

Another result determined that the participant decision to combine different treatments is not related to the number of medicinal plants known locally for dealing with a disease. Since decision making tends to be guided by personal perceptions [45], the lack of relationships between these variables is possibly due to the mismatch between the total number of medicinal plants available in the community to deal with a disease and the number of medicinal plants known by each person. A study by Santoro et al. [14] in rural communities in Northeast Brazil showed that only a few medicinal plants are well shared. The same pattern may be happening in the studied community.

Regarding the socioeconomic factors evaluated, only education showed relationships with simultaneous use. The relationship between local knowledge and education is controversial in ethnobotanical studies, because there is empirical evidence for positive, neutral and negative effects [26]. However, the majority of studies that evaluate the effect of education cite evidence of the negative effect over local knowledge [26]. One of the reasons for that finding is that residents with higher educational levels spend more time learning academic skills than local practices [46]. In addition, education could also be considered a covariate of income and occupation rather than the main explanatory factor [47].

In this study, we propose that education may have another role in the combined uses of plants and modern medicine. In the community studied, to advance in formal educational studies, residents need to commute to the urban area of the municipality. This change in environments may expose these residents to additional contact with biomedical practices, which could facilitate the copying of such information.

Another factor to consider is that information compatible with other information previously known by residents tends to be more efficiently copied in human populations [48]. This factor may explain why the presence of the biomedical system may be rejected in some local medical systems, since the concepts of health and disease in the two systems are not the same. However, additional years of formal education may enable residents to better understand the concepts of a cosmopolitan medical system, which may facilitate the absorption of such practices and the copying of such information.

The other socioeconomic factors evaluated in this study (age, sex, and local prestige) did not show any relationship with the combination of treatments. A study by Waldstein [20] showed that Mexican migrant women living in the United States used medicinal plants in combination with modern medicine. However, they relied primarily on their plant treatments. In their responses, some residents mentioned that they used the two resources but relied more on the use of plants. The lack of this relationship observed in this study might be because the present study only investigated whether or not residents used modern medicine in combination with medicinal plants and not the preferences in those cases. Therefore, this area may be an interesting research topic for future studies.

## Conclusions

The combined use of modern medicine and medicinal plants follows adaptive patterns in local medical systems. This study shows that frequent, severe and chronic diseases promote more combined use, demonstrating that the introduction of biomedical treatments is facilitated at critical points in the system, where healing is more required. Thus, the necessity to survive causes residents to adopt new treatments in local medical systems. Possibly, this same pattern facilitates the adoption of other external health practices, such as the use of exotic plants species in local pharmacopeia. This issue needs to be addressed in future studies.

The coexistence of the two kinds of health practices (local and cosmopolitan) enhances health care capacity in rural communities. The presence of governmental health programs in rural areas contributes to disseminating biomedical practices. The combination of treatments may reflect the preservation of local knowledge, since residents adopt new practices but together with the local cures. However, our study does not evaluate the resident preference for one treatment or another. We neither address if combined use is the first therapeutic strategy or follows the ineffective use of medicinal plants or modern medicine. These are limitations of our study, in that we cannot speculate about the resistance of residents in adopting other external healing practices.

Among socioeconomic factors, education was the only factor that influenced the combined use. Access to formal education can increase contact with and facilitate understanding of how the biomedical system works, increasing the learning of this information. However, age, sex and local prestige did not influence the combined use. These factors may not have had any effects in the present study because the need for a cure is a strong selective pressure that favors the introduction of external medical practices. However, we need to replicate our study in different human populations to understand whether this is a particular feature of the investigated community or a general pattern.

## Supporting information

S1 File Data set used for statistical analysis. (CSV)

S2 File R scripts used in the statistical analysis. (DOCX)
