# Supplementary material for: Factors in hybridization of local medical systems: Simultaneous use of medicinal plants and modern medicine in Northeast Brazil
Source: PLoS One. 2018 Nov 14;13(11):e0206190. doi: 10.1371/journal.pone.0206190 (PMC6241117; doi:10.1371/journal.pone.0206190)
Supplement: S2 File — (DOCX) [file pone.0206190.s002.docx]

**R scripts used in the Statistical analysis**

#*Packages used for the statistical analysis*

library(lme4)

library(lmerTest)

library(sjPlot)

#*Importing the data set*

Data<-read.csv2("S1.csv", h=T)

str(Data)

#*Creating the null model*

Nullmodel<-glmer(Use_overlap~ 1 + (1|Participants_ID),family="binomial",control=glmerControl(optimizer="bobyqa"), Data)

summary(Nullmodel)

#*Models using functional variables*

Model_1<- glmer(Use_overlap~ Disease_frequency + Disease_severity + Disease_manifestation + Utilitarian_redundancy + (1|Participants_ID),family="binomial",control=glmerControl(optimizer="bobyqa"), Data)

summary(Model_1)

Model_2<- glmer(Use_overlap~ Disease_frequency + Disease_severity + Disease_manifestation + (1|Participants_ID),family="binomial",control=glmerControl(optimizer="bobyqa"), Data)

summary(Model_2)

Model_3<- glmer(Use_overlap~ Disease_frequency + Disease_severity + (1|Participants_ID),family="binomial",control=glmerControl(optimizer="bobyqa"), Data)

summary(Model_3)

Model_4<- glmer(Use_overlap~ Disease_frequency + Disease_manifestation + (1|Participants_ID),family="binomial",control=glmerControl(optimizer="bobyqa"), Data)

summary(Model_4)

Model_5<- glmer(Use_overlap~ Disease_severity + Disease_manifestation + (1|Participants_ID),family="binomial",control=glmerControl(optimizer="bobyqa"), Data)

summary(Model_5)

# *Testing the validity of the explanatory model*

anova(Nullmodel,Model_1)

#*Models using socioeconomical variables*

Model_6<- glmer(Use_overlap~ Schooling_category + Age + Sex + Prestige + (1|Participants_ID),family="binomial",control=glmerControl(optimizer="bobyqa"), Data)

summary(Model_6)

Model_7<- glmer(Use_overlap~ Schooling_category + Age + Sex + (1|Participants_ID),family="binomial",control=glmerControl(optimizer="bobyqa"), Data)

summary(Model_7)

Model_8<- glmer(Use_overlap~ Schooling_category + Age + (1|Participants_ID),family="binomial",control=glmerControl(optimizer="bobyqa"), Data)

summary(Model_8)

Model_9<- glmer(Use_overlap~ Schooling_category + (1|Participants_ID),family="binomial",control=glmerControl(optimizer="bobyqa"), Data)

summary(Model_9)

# *Testing the validity of the explanatory model*

anova(Nullmodel,Model_6)

#*Model combining the most important functional and socioeconomical vriables*

Model_10<- glmer(Use_overlap~ Schooling_category + Disease_frequency + Disease_severity + Disease_manifestation + (1|Participants_ID),family="binomial",control=glmerControl(optimizer="bobyqa"), Data)

summary(Model_10)

# C*omparing the best fit model of functional variables with the integrative model (functional and socioeconomical variables)*

anova(Model_2,Model_10)

#*Graphs*

sjp.lmer(Model_1, type = "fe", axis.lim = c(-1, 1))

sjp.lmer(Model_6, type = "fe", axis.lim = c(-2, 2))

sjp.lmer(Model_10, type = "fe", axis.lim = c(-1.5, 1.5))
